# Do bone scans add to CT in detecting skeletal metastases in breast cancer staging?

**DOI:** 10.1186/bcr3798

**Published:** 2015-11-05

**Authors:** Anuradha Anand, Lekha Potti, Geoff Naisby, Sheetal Sharma, Alan Redman

**Affiliations:** 1 James Cook University Hospital, Middlesbrough, UK; 2 Queen Elizabeth Hospital, Gateshead, UK

## Introduction

Recent studies have questioned the value of bone scans (BS) in staging breast cancer when a CT chest, abdomen and pelvis is also performed. We retrospectively reviewed breast cancer staging CTs and BS performed within 2 months of each other, to see if BS identified more skeletal metastases than CT.

## Methods

Our study was performed at the breast screening unit at Queen Elizabeth Hospital, Gateshead (QE) and the symptomatic breast unit at James Cook University Hospital (JCUH), Middlesbrough. Experienced radiologists blinded to primary BS reports retrospectively assessed CTs performed for primary breast cancer, known recurrence or to explain symptoms of pain. They then reviewed the same patient's BS. CT and BS were marked positive, negative or indeterminate for skeletal metastases.

## Results

Combined data from both units yielded 253 cases in total. CT and BS concurred in 217 cases. Of the remaining 36, CT identified skeletal metastases in five where BS was negative and two where BS was indeterminate. CT excluded metastases in 23 which were indeterminate on BS. BS confirmed or excluded metastases in five cases where CT was indeterminate and identified metastases in only one case which was negative on CT. This lesion proved to be benign and hence BS was false positive in this case.

## Conclusion

BS does not detect more skeletal deposits than CT in the initial assessment or follow-up of breast cancer. CT should be used as the first-line investigation for skeletal and visceral metastasis and BS reserved for problem-solving.

